# Health and Greater Manchester in Historical Perspective

**DOI:** 10.1080/00344893.2016.1165513

**Published:** 2016-03-30

**Authors:** Stephanie J. Snow

## Abstract

This article maps the history of health organisation across Greater Manchester (GM), primarily since the Second World War, to show how against a continuing backdrop of health inequalities, services have been driven (and constrained) by the needs and the politics of each period. Defining ‘success’ as benefits for patients the article identifies examples such as Salford’s mental health services (1950s and 1960s), public health in North Manchester (1970s and 1980s), the creation of centres for diabetes, sickle-cell and thalassaemia (1980s) and the formation of the Joint Health Unit in 2002. What this history shows is that over the period the common factors influencing the ‘success’ of health organisation across GM have been the championing of particular issues by multi-disciplinary groups working across health and social care and stability in structures and personnel.

## Introduction

Since its rapid urbanisation from the 1750s onwards, Manchester and the surrounding metropolitan boroughs that together make up Greater Manchester (GM), have been paradigmatic for health inequalities. At the end of the nineteenth century the poor physique of Manchester men caused a national scandal when three out of every five volunteers for the Boer War were deemed unfit (Rodger 1995: 54). Today, the health of GM people remains worse than the England average with higher than average levels of deprivation and over 30% of children living in poverty. Life expectancy for men and women is lower than the England average and on many measures including teenage pregnancy, smoking, obesity, sexually transmitted diseases, tuberculosis, cardiovascular disease and cancer, the health of GM compares poorly to other parts of the country (Manchester Unitary Authority 2015). GM health inequalities thus seem intransigent across time and social context despite evidence that many inequalities in health are not ‘inevitable’ and can be reduced (Marmot 2010: 29). Yet the history of health organisation in GM is far more diverse and features innovation and achievements that have benefited patients, as well as difficulties and failures. ‘Devo Manc’ is probably the most ambitious initiative in GM’s health history to date. But how does this history speak to the present? And how can it contribute to delivering local ambitions for ‘Devo Manc’? This article offers a broad overview of the history of health organisation across GM focusing primarily on the period since the Second World War. It uses vignettes of different periods and services to show how developments have been driven (and constrained) by the needs and the politics of each period. What we learn from this history is that over the period the common factors influencing the ‘success’ of health organisation, defined for the purpose of this article as benefits for patients, have been local champions who have promoted cross-working and collaboration across health and social care organisations, aided by continuity in structures and personnel.

## Industrialisation and Health

Manchester’s first hospital was established in 1752 by the surgeon, Charles White, who had trained in London with William Hunter, pioneer in the field of obstetrics. Funded by local philanthropists and subscribers, the infirmary proved highly successful and by the mid-1790s was one of the most comprehensive systems in Britain. It treated in-patients, out-patients, provided home visits, developed an asylum and created the first fever hospital in Britain to treat victims of epidemics such as typhus fever (Pickstone 1987). Many more charities developed during the first half of the nineteenth century including dispensaries in Ancoats and Salford which later became full hospitals. But the health of the poor working classes led to Manchester developing a reputation as the shock city of the nineteenth century. The best-known critique of the social conditions in Manchester was written by the German philosopher, Friedrich Engels, whilst visiting the city during 1842–44. He worked at the family-owned mill, Ermen and Engels at Weaste, Salford and spent his leisure time visiting poor working-class communities. Off Oxford Road on the south side of Manchester Engels found 40,000 Irish immigrants living in abhorrent conditions of filth and stench. Through his observations he established that mortality from infectious diseases such as fevers, smallpox, measles and whooping cough was four times higher than outlying rural areas and that mortality rates were significantly higher than the national average. He directly related these stark health indicators to the appalling living and working conditions of workers which were driven by capitalist enterprise: ‘Women made unfit for childbearing, children deformed, men enfeebled, limbs crushed, whole generations wrecked, afflicted with disease and infirmity, purely to fill the purses of the bourgeoisie’ (Engels 1987: 171–84). Despite doctor-led initiatives such as the Manchester and Salford Sanitary Association (1852), Manchester was a late starter on the public health front, not appointing its first Medical Officer of Health (MOH) until 1868: Liverpool appointed William Duncan as MOH in 1847. The advantages for the local population arising from these public health pioneers are well-illustrated through the work of one of the longest serving MOHs, James Niven, who was appointed MOH for Oldham in 1886 and moved to Manchester in 1922. Whilst in Oldham Niven improved housing standards, sewage and refuse disposal, milk and water supply, reduced smoke pollution and contained the spread of infectious disease. He is credited with reducing the death rate in Manchester from 24.26 per 1000 population in 1893 to 13.82 per 1000 population in 1921 through an ‘intense zeal, perseverance and courage of conviction’ that drove sanitary reforms and waged war on infectious disease (Elwood and Tuxford 1984: 99). In part his success derived from a willingness and ability to develop cooperation between Manchester and other authorities. To coordinate housing reform across the region, for example, Niven had to liaise with over a hundred other sanitary authorities within 15 miles of Manchester to ensure that town-planning, main roads and transport arrangements dovetailed (BMJ 1923: 468). Since industrialisation the region has incorporated a multiplicity of boroughs, each with its own system of local government and the enduring challenge has been (and remains) the coordination of healthcare planning and provision across the different geographies and communities. By the 1930s, Manchester and Lancashire County Council were regarded as leaders in health and social integration and the willingness of the local authorities and health organisations to work collaboratively set a national precedent for the joint planning of health services (Pickstone 1987). In part this was driven by a local outcry about the death of a young Jewish mother, Mrs Molly Taylor, who had been transferred between hospitals an hour after giving birth.

## Joint Planning and the National Health Service

On 12 May 1934, Mrs Taylor presented in labour to St Mary’s Hospital, gave birth in the out-patients and was rapidly transferred to Crumpsall Hospital where she died the following day. The cause of death was agreed to be delayed obstetrical shock which could not have been foreseen and was unlikely to have been induced by the transfer. Nevertheless the public inquiry in September 1934 revealed a chain of poor care: there was no space on the labour wards at St Mary’s as a fifth of the total beds were unusable due to spring-cleaning so Mrs Taylor had to give birth in the out-patients’ room; she was not given food or drink after the birth; the nurse who accompanied her in the ambulance was inexperienced; and she was not medically assessed on arrival as Crumpsall had no residential obstetrical doctor(Pickstone 1985: 275–6). The Ministry report on Manchester Public Health Services exonerated them from causing the death but recommended changes to better coordinate care across services. The result was the creation of the Manchester Joint Hospitals Advisory Board in 1935; reconstituted as the Manchester Salford and Stretford Joint Hospitals Advisory Board in 1942. The Board was chaired by Sir Christopher Needham, chairman of the University of Manchester Council, and members included local councillors, Medical Officers of Health and medical consultants; in 1936 local general practitioners (GPs) joined the Board. It had three key foci: the coordination of hospitals serving the same catchment area; the distribution of specialist care from the major voluntary hospitals to other general hospitals; and the development of new regional services. The hospitals remained under separate management but were grouped into North, Central and South, taking into account the complexities of geography and dynamics of medical training; training posts in the teaching hospitals were more attractive appointments than the municipal hospitals which had their origins in workhouse hospitals. The Board began to collect information on hospital waiting lists and to plan the development of specialist services. During the inter-war period Manchester developed new specialties including orthopaedics, pioneered first at Ancoats Hospital by Harry Platt (Anderson, Neary and Pickstone 2007). By the 1930s orthopaedics had been taken up by the Manchester Royal Infirmary (MRI) and one of the first fruits of the Board was the building of a new orthopaedic block in 1936 which was opened by Walter Eliot, Minister of Health. Hailed as ‘the first concrete, or rather ferro-concrete result’ of the Board, Eliot praised the remarkable work being undertaken which served as a national example for effective coordination between the voluntary and statutory sectors (Pickstone 1985: 287).

During the Second World War, local cross-working and collaboration fed into the success of the North West Emergency Medical Service which, unlike most other areas, was run on a regional basis (Pickstone 1985: 296–7). Support for health service reform and the expansion of services by bringing all hospitals under local authority control grew during the war. But the surprise Labour election win in 1945 brought in its wake a new Minister of Health, Aneurin Bevan. GP and consultant opposition to the prospect of becoming salaried state employees led to Bevan setting the old plans aside. His solution was to create a National Health Service (NHS) by nationalising all hospitals and grouping them locally according to function and type (Berridge 1999: 15–8). Administration was through a hierarchy of regional, group and hospital management committees with members drawn from doctors, philanthropists and councillors. Regional hospital boards reported to the Minister of Health and the first chair of the Manchester Regional Hospital Board was Sir John (later Lord) Stopford, then Vice Chancellor of Manchester University. Teaching hospitals were exempt from these arrangements and retained their Boards of Governors, reporting directly to the Minister of Health. Local authorities retained jurisdiction over public health covering mothers and children; midwifery; health visiting and nursing; vaccination and immunisation; ambulances; mental health; health education and sanitation. GPs remained independent professionals, contracting services into the NHS.

The North West topped the headlines on 5 July 1948 when Bevan declared the opening of the new NHS at Park Hospital, Davyhulme, renamed Trafford General Hospital in 1988. Despite gloom amongst MOHs that the new structures had significantly diminished the role of local authorities in health, Charles Metcalfe Brown, MOH for Manchester, delighted in the new opportunities. As preventive medicine improved in efficiency and results, he argued, so the need for therapeutic medicine would diminish and thus the importance of public health would grow. One of the key changes under the NHS was the organisation and administration of mental health services and Salford led the way in rethinking mental health service provision.

## Salford Mental Health: A New Vision of Community Care

The new NHS came at a time of significant change for mental health with new ideas about treatments and the beginning of an overarching (and continuing) policy shift to treating the mentally ill in the community (Busfield 2001). Under the NHS, responsibility was divided for mental health services across each region: local authorities had care of clinics and social workers (although their functions were permissive) and mental hospitals were overseen by the regional hospital boards. Most hospital admissions were compulsory but there were no catchment areas and patients with recurring illness could be admitted to different hospitals on successive occasions thus losing any continuity of care. Until 1956 most Salford patients went to Prestwich Hospital which had over 3000 beds but it functioned as a closed system and had little working contact with local authority services. The new structures caused services to become less integrated than in previous decades (Welshman 1999: 208). The NHS gave all citizens the right to care from a GP and this introduced the possibility of earlier diagnosis of mental health problems (Rehin and Martin 1968). Unlike many parts of the UK, Salford was particularly well-placed to capitalise on these new opportunities as it had a dynamic MOH: J. Lancelot Burn had been appointed to the public health department in 1941. Mental health was one of his priorities and he was unusual in his view that the community, rather than an institution, was the natural care environment. Before 1948 he had set up a mental health sub-committee with a brief of creating a bridge between hospital and community. But the tripartite structure of the NHS perpetuated historical and cultural differences, producing duplication and conflicts of interests between organisations concurrently caring for the same patient. Burn’s focus on two main principles: appointing well-trained staff and developing collaborative working across services, proved the key to breaking down boundaries (Harrington 2008: Chapter 2).

Burn developed community resources such as a therapeutic social club, a women’s day centre and collaborated with the University of Manchester’s Department of Social and Preventive Medicine (Harrington 2008: 76). He had the advantage of good funding and strong support from Salford’s Labour Council but the main challenge was changing working practices and culture. In 1957 he appointed Mervyn Susser, a South African epidemiologist, to lead the mental health department and this proved a pivotal appointment. Susser’s early initiatives included organising Mental Welfare Officers around GP practices so that each GP had a known officer; sending copies of patients’ progress reports to GPs; establishing weekly team meetings to develop skills and promote new attitudes of cooperation between doctors and social workers; and probably most importantly, developing a new consultant post with responsibilities across general and mental hospitals, out-patient clinics, and local authority services. Hugh Freeman, psychiatrist, was appointed to this post in 1961. Born in Salford in 1929, Freeman was strongly aware of the poverty of the area and the ‘political dimension’ of medicine (Harrington 2008: 99). He pioneered psychiatric units in general hospitals at Hope Hospital and Salford Royal, built up day and out-patient care, and developed multi-disciplinary teams of mental health social workers, mental welfare officers, nurses and GPs so that patients could be treated in community settings. He established one of the first registers for psychiatric cases and used this to monitor the population’s needs around mental health. By 1968 Salford was recognised for its ‘excellent programmes’ that were distinguished by their being driven by the local authority rather than the hospital (Harrington 2008). Manchester had adopted a strategy of developing psychiatric services from within the general hospitals in Oldham, Bolton, Burnley and Blackburn (Freeman 1984). But wider changes beyond Salford in combination with local unrest were to disrupt the status quo.

Since 1948 Salford had enjoyed staffing levels and funding levels that were well above national averages and this had enabled the service development. But this era was brought to an abrupt close when in 1968, Labour lost control of Salford Council to the Conservatives. The new Council cut the health committee budget and two of Salford’s support centres—Cleveland Day Centre and Kersal House—faced closure. The department launched a strong public campaign which gained national press coverage and Frank Allaun and Stanley Orme, two local Labour MPs, lobbied Richard Crossman, Secretary of State for Social Services, who intervened directly to save the centres. Nevertheless, several posts were frozen and the funding reduced. During the same period Burn retired from his post and within 12 months, George Mountney who had been the mental health officer in Salford since 1955 noted the detrimental consequences on services: ‘The effect of losing the staff is that we regress to the type of service that we provided in the past … a service oriented to deal only with psychiatric crises and emergencies’ (Harrington 2008: 125). And more disruption was to follow with the reorganisation of social services following recommendations of the Seebhom Committee, appointed in 1965 to review social services functions of local authorities. On 1 January 1971 Salford’s mental health department was transferred to Salford Social Services. Mental health social workers were allowed to keep their specialist functions in the new department but over time the networks between the community and hospitals weakened and the difficulties were compounded by the 1974 NHS reorganisation which redrew the health service and local authority boundaries causing Salford to double in size and integrate its social services with Lancashire. Thus what had been an exemplary provision of acute and community services for mental health suffered collapse within a matter of months. By 1971 there was evidence of a measurable drop in the quality of support for users of the services (Harrington 2008: 130).

## North Manchester: From Crisis to the Best of Health

The 1974 NHS reorganisation was designed to improve coordination of health and social services by dovetailing health and local authority boundaries hence the integration of parts of Lancashire services with Salford. But as Webster noted: ‘The paradoxical consequence … was a service more bureaucratically complex and only marginally more unified’ (Webster 1993: 130). The North Western Regional Health Authority (NWRHA) covered a population of 4.1 million and had 11 Area Health Authorities (AHA) under its control. AHAs took over responsibility for many of the health services previously managed by local authorities including vaccination, health centres, family planning, school health, health visiting and home nursing. It brought radical change to public health as MOHs were replaced by Community Physicians who were attached to the different levels of the NHS (Lewis 1986). Local authorities retained responsibility for environmental health, communicable diseases, housing, clean air, pollution, food safety and pest control. More widely, the growing costs of the NHS in the context of the 1970s economic crises put extreme pressure on services and community health remained a low priority. Significantly this reorganisation decentralised services in Manchester by creating three separate geographical districts: North, Central and South. This had long-lasting negative consequences as it encouraged districts to adopt a parochial approach to services rather than taking a Manchester-wide approach. There is evidence that this arrangement had been encouraged by the NWRHA as it wanted to avoid the potential difficulty of having a very ‘strong’ Manchester if it remained as one unit (Jones and Pickstone 2008: 59). The problematic structures led to a further reorganisation in 1982 which removed Areas and gave direct responsibility for defining and developing health services to District Health Authorities (DHA). With major teaching hospitals, Central and South Districts benefited from the policy priority accorded to acute services whereas North District had histrically suffered from a lack of investment and some of the worst health inequalities across the city. Yet during this period and without significant financial investment, North Manchester DHA and its Public Health Department developed new approaches to service development.

Underpinning the achievements in North Manchester was the leadership of Professor Joe Moore, Chair of the North Manchester DHA Board and Chief Executive, Mike Brown, who worked to build good relations with the Region and create an understanding of the significant challenges posed by the district’s health inequalities (Jones and Pickstone 2008: 63–4). It was not an easy period as Moore and Brown led a rationalisation programme requiring the closure of Ancoats, the Jewish hospital and the former isolation hospital at Monsall. The monies saved were used to fund public health and provide community-based alternatives to hospital care through close working with local GPs. At the same time, a major hospital improvement programme was begun at North Manchester General, funded by a loan from the NWRHA. Moore was a regular presence in the area, visiting hospitals and clinics and this improved the historically difficult industrial relations with NHS workers. Community Health Councils had been created as part of the 1974 reorganisation to provide public representation at district level and Moore became a regular attender at local meetings. The process of open dialogue and opening DHA meetings to the public helped resolve local opposition to change. In the case of the proposed closure of Ancoats A&E for example, Moore and his team identified the key issues causing concern to local clinicians and the public and addressed these by setting up a walk-in-clinic to treat minor injuries and making ambulance transport available for walk-in patients requiring emergency treatment (Jones and Pickstone 2008: 67–8). In 1988 Brian Deer, the first journalist to specialise in social affairs at the *Sunday Times*, ran ‘The Best of Health’ competition supported by the National Association of Health Authorities, the Institute of Health Service Management, the NHS Management Board and the PA Consulting Group. North Manchester Health Authority was the winner and praised for its success in developing new services in the context of difficult economic conditions (Deer n.d.). Indeed, Ken Jarrold, General Manager of Gloucester Health Authority and a runner-up commented: ‘The good thing about North Manchester winning is that nobody in the NHS can say they could not do the same’ (*Sunday Times* 1988).

One advantage North Manchester had that was certainly not shared in other Manchester districts, nor in many places across the UK was continuity in staff. In 1982 Dr Joyce Leeson, previously District Medical Officer in the South, moved to the North district, remaining there until the 1990s. Leeson was a political radical and a feminist and had worked in the University of Manchester’s Department of Social and Preventive Medicine (Jones and Pickstone 2008: 63). She developed an ethos of close working with the local authority, the local community and voluntary organisations. The benefits of this approach are exemplified in the achievements of the Women’s Health Team. Leeson had set up a Well Women Clinic whilst working in South Manchester (Williams 1987: 107–28) and in 1985 together with Judith Gray, Community Physician at North Manchester General Hospital, she set up the North Manchester Women’s Health Team. This was a first of its kind, embedded in the NHS but offering a radical approach to preventive care and manifesting the beliefs of the wider women’s health movement (Williams 1987: 108–10). Rather than the medical model found in other parts of Manchester where Well Women Clinics were run by health visitors or clinicians, the North Manchester ethos involved other workers, agencies and local women in the planning and development of services. Hard to reach groups like older women, disabled women, Asian women and young women were identified and efforts made to identify the sorts of services that would appeal to these various needs. The setting up of a Well Women Centre in the Cheetham Hill area of North Manchester was the result of campaigns by local women workers and activists representing the large non-white population. An Asian women’s health worker was appointed to coordinate outreach and service development and Neesa, a drop-in centre offering health and social support to women was created. The creation of a District Planning Group for Women’s Health Services ensured that women’s health needs were embedded and taken account of in strategic planning (Jones and Pickstone 2008: 65). Legacies remain from this period as Neesa continues to operate as a community resource (Neesa). North Manchester’s success during this period was due to a combination of strong local leadership supported by stability in organisational structure and personnel.

## Central Manchester: Innovation, Chronic Disease and Communities

The election of the Thatcher government in 1979 had heralded an era of ‘continuous revolution’ in the NHS (Webster 2002: 144). Major funding pressures led to a raft of initiatives designed to make the NHS more efficient and accountable and included the introduction of general management in 1983, notable for being the first serious attempt to shift the ‘frontier of control’ between doctors and government (Harrison and Lim 2003). The creation of the Manchester Diabetic Centre (MDC) at the MRI in 1988 during a period of intense financial pressure illustrates how clinicians willing to engage with the ethos of the period could deliver innovations for patients that also pleased managers.

The MRI had provided a diabetes clinic since the 1920s following the Canadian discovery of insulin therapy which had transformed diabetes from an acute and terminal illness to a chronic condition. By the 1950s the clinic had a reputation of being the ‘best’ but attracted so many patients that there were long waits and little continuity of care (Valier and Bivins 2002: 41). The refinement of insulin, more effective injection methods and better control of secondary complications through the 1960s and 1970s all contributed to improved treatments. Nevertheless, diabetes was not generally taken seriously within acute care despite the very high costs associated with diabetic patients. In 1984 Stephen Tomlinson who had an established interest in diabetes was appointed Professor of Medicine by the University of Manchester and inherited the diabetes clinic (Valier and Pickstone 2008: 68–9). Under-funding and rising attendance figures combined to create conditions where patients could wait four hours to see a doctor for five minutes or less. Tomlinson found this highly unsatisfactory and undertook to change the ‘doctor-dominated’ culture of diabetes services (Valier and Bivins 2002: 44). He was strongly influenced by the national diabetes community, particularly the work of the South East Thames Diabetes Physicians Group and the British Diabetic Association (BDA) which sought reforms such as patient education, GP co-operative care schemes and support from specialist diabetes nurses. The decision to build a new centre away from the MRI was driven by a desire to ‘engage *directly* with the people who have the problems’ (Valier and Bivins 2002: 44). Tomlinson sought support from all parties including the DHA, the hospital management, pharmaceutical companies, local industry, the BDA and patient groups and was highly successful in fundraising. The MDC on Hathersage Road at the southern end of the Oxford Road site opened five days a week and Tomlinson defined its primary objective as ‘prevention and self-care through education’. It would be achieved, he argued, through abandoning the ‘problem-finding environment’ of the traditional diabetic clinic with a new preventive approach (Valier and Bivins 2002: 45). He was supported by Jill Pooley, originally appointed in Stockport as one of the first diabetes specialist nurses in the country but then headhunted by Tomlinson to plan and fundraise for the MDC. Pooley’s sensitivity to patient needs and experiences were critical in shaping the direction of the MDC. The role of the diabetic nurse was developed to enable nurses to undertake patient consultations and deliver patient education programmes. They were supported by other paramedics such as chiropodists and dieticians and thus delivered a one-stop shop for diabetic care. Tomlinson promoted heavily the economic benefits of the MDC arguing that the new way of providing care would prevent secondary conditions. Diabetic foot, for example, was the commonest cause of in-patient admission and thus very costly but Tomlinson estimated that between 50% and 80% of diabetic amputations were preventable if early treatment was available. The only resistance to the MDC came from GPs who viewed it as further evidence of resources being allocated to hospitals rather than primary care. To counter opposition, Tomlinson and Pooley established mini-clinics located at GP premises and a purpose-built centre in Bolton. Tomlinson audited the outcomes of the MDC and mini-clinics which allowed him to predict reductions in in-patient admissions and consequent cost-savings (Valier and Bivins 2002: 46–7). This brought to light the lower rates of attendance from Manchester’s South Asian communities and the MDC responded by introducing clinics with an Asian nurse and co-opting the Manchester Action Committee for the Health Care of Ethnic Minorities (MACHEM) to assist in the process.

Developing health services that met the needs of Manchester’s growing multi-ethnic communities was one of the biggest challenges of this period. Manchester had a long history of providing homes and work for a multiplicity of nationalities and ethnic groups and became a major centre for migrant communities from the 1950s onwards (Jones and Snow 2010: 23–32). Indeed, thousands of doctors and nurses had been recruited from across the world to sustain and develop GM health services since the creation of the NHS in 1948. The particular challenges facing the NHS in responding to such a large immigrant community were raised by Central Manchester CHC in 1976. Linguistic difficulties, vitamin deficiencies and the psychiatric needs of those who had moved from a ‘peasant’ to an industrialised society were all identified in its Annual Report (Manchester Central Community Health Council Annual Report 1975–1976: 12). Over time and with the involvement of the ethnic communities, translation support and services targeted at the specific health needs of migrant groups were introduced. Most communities settled in the inner city creating a pattern of residential segregation as more affluent groups moved to live in the southern suburbs and other outlying areas. Thus by the 1980s, the most impoverished areas of the city housed black and Asian residents (Jones and Snow 2010: 26). One of the most successful initiatives was the development of sickle-cell anaemia and beta-thalassaemia services through the establishment in 1984 of the Manchester Sickle Cell Centre in the Moss Side area, home to a large African-Caribbean community (Valier and Bivins 2002: 52–6). Sickle-cell anaemia is a rare inherited blood disease which in the UK mainly affects people of African and Caribbean origin. There is no cure for the condition and so in the 1980s, as now, care focused on patient education, screening and education. The creation of the Centre was facilitated with the help of the Moss Side Family Advice Centre which had been set up in 1973 to support young black and Asian immigrants and help alleviate racial tension. Voluntary research showed low public awareness of sickle-cell anaemia. Representations to the Rev Dr Peter Povey, director of public health in Manchester, led to the establishment of a working group including members from the Manchester Central CHC, the Community Health Group for Ethnic Minorities, and Verna Davis from the Regional Health Promotion Unit. The NWRHA was eventually persuaded to fund a two-year programme of research to evaluate the number of sickle-cell sufferers in GM, the sorts of services required and what was already being provided. The key finding was the need to establish a drop-in community service embedded in the local, high-risk population. Further investigation revealed a high local incidence of thalassaemia, another inherited blood disorder which required genetic counselling. The Centre worked with MACHEM to establish local counselling services. The result was the appointment of Dr Rafeya Rahman in 1991 and the renaming of the Centre as the Sickle-Cell and Thalassaemia Centre. Huge local support for the services voiced through a public meeting at Manchester Central Library ensured continuance of funding from the Manchester Health Authority and a growing national recognition of the need for such services guaranteed their longevity (Valier and Bivins 2002: 55). The strong local profile of the Centre enabled it to raise funds from the local business community and teaching on these diseases has now been integrated into the medical curriculum (Manchester Sickle Cell and Thalassaemia Service).

## Towards Cross-working: Healthy Cities, Health Action Zones and the Joint Health Unit

Since the creation of the Joint Hospitals Advisory Board in the pre-NHS era there have been many attempts to improve coordination of healthcare across GM and between the various authorities. The Local Government Act (1985) abolished the Greater Manchester Council in 1986 and devolved power to local areas. The Association of Greater Manchester Authorities was created as a partnership between the area’s ten local authorities and in 2011 formed into the Greater Manchester Combined Authority, the first of its kind in the UK. Nevertheless, the challenges of developing long-term local strategies in the face of political cycles and short-term policy change have been significant. The early 1990s brought new organisational upheavals with the introduction of a quasi-internal market which split purchasing and provision functions as a means of increasing competition and improving efficiency. Trust structures created self-governing organisations that were obliged to market their services to the new purchasers and meet national targets and performance indicators. In primary care, a voluntary scheme was introduced giving GPs the option of becoming ‘fund holders’ with budgets for purchasing secondary care directly from providers. The introduction of GP fundholding (GPFH) was a key moment in the history of general practice as, for the first time, GPs’ powers as GPFH enabled them to challenge the provision and delivery of hospital services producing a paradigm shift in relationships (Pollock 2005: 141). GPFH promoted new intra-professional networking which improved services for patients in some areas. In Stockport, in part as a result of the networks formed by fundholding GPs working together, GPs formed cooperatives across localities to provide out of hours cover. But much focus remained on acute care and the ongoing rationalisation of services across the region which required hospital closures and the designation of single sites for tertiary specialties like children’s services and neurosciences inflamed an already difficult relationship between the City Council and Manchester Health Authority (Jones and Pickstone 2008: 92). The Conservative White Paper *The Health of the Nation* (1992) which set targets for disease and mortality reduction in key areas such as coronary heart disease and mental health, advocated cross-working. This seemed perverse in the context of an internal market. Leeson (1991) asked:

We are told that the great opportunity for public health doctors in the purchasing authority will be to demand and specify quality in contracts. Will it be possible to address inequalities by involving communities inter-sectoral work, and ensuring the creation of quality services like our HIV/AIDS services just by setting contracts? (Leeson 1991)

In 1987 through the Healthy Cities Initiative, a European network designed to promote urban health, Manchester had established a Health for All working party including the City Council, the Health Authorities and the voluntary sector. Two new Council posts were created for a health campaign worker and a public health doctor specialising in housing, health and health inequalities, and this laid the basis for the setting up of a Health Promotion Unit in 1987. During this period, there were pockets of success as in North Manchester’s response to HIV/AIDS which was a strong example of ‘collaborative work involving health promotion, AIDSline and local authority staff working together with no independent budget’ (Jones and Pickstone 2008: 77–8) but no significant citywide gains: Manchester did not win Healthy City status until 1996.

Health inequalities were given increased priority through the New Labour government elected in 1997 with the creation of Health Action Zones (HAZ). Manchester, Salford and Trafford Health Authorities and the three Councils were successful in their bid to establish a HAZ. Out of the 26 HAZs created, Manchester was ranked second to bottom by health and deprivation (Department of Health 1999: 13). HAZs were designed to create local freedom and stimulate community-led initiatives but over time the national focus on performance management reduced the ability of the HAZ to deliver. There were some successes such as the Wythenshawe Health Strategy emergency contraception scheme in cooperation with local pharmacists and work around tobacco control. Importantly the HAZ brought health inequalities into new focus and the partnership building produced important legacies such as the creation of a social enterprise to fill workforce gaps in the care market (Jones and Pickstone 2008: 97).

Like its predecessor governments, New Labour did not desist from reorganising the NHS and in 2002 Regional Health Authorities transformed into Strategic Health Authorities (SHA) and Primary Care Trusts (PCTs) replaced Health Authorities. North, Central and South Manchester PCTs were overseen by the GM SHA but in 2006 a further reorganisation created a single Manchester PCT and merged GM with surrounding SHAs to create the North West SHA. National policy during this period emphasised the importance of collaboration and partnership and here Manchester was ahead of the game. The Manchester Joint Health Unit (JHU) was formed in April 2002 in response to concern that creating three PCTs would diminish focus and collaboration on citywide initiatives. The JHU was informed by a report commissioned from Durham University that linked the ‘unacceptable’ health inequalities in Manchester to the lack of Council involvement in local health priorities and the need for better understanding and coordination (Jones and Pickstone 2008: 108). The JHU, based in the Town Hall, was funded by the Healthy City fund, the City Council and the Manchester PCTs. It has promoted many public health improvements that required cross-working including the Valuing Older People initiative which developed successful and innovative programmes around ageing in collaboration with the Manchester Institute for Collaborative Research on Ageing at the University of Manchester. In April 2014 Manchester was recognised by the WHO as the first Age-Friendly City in the UK (Handler 2014).

The JHU can be claimed as successful on many levels but the history of cross-working in Manchester speaks to the difficulties of achieving objectives when organisational structures and boundaries between health and social authorities have frequently changed. Evidence of the advantages conferred by continuity in structures and personnel within GM can be found in the example of Salford and Stockport (Snow 2013). Since 1974 Stockport Health Authority and its successor organisations have been coterminous with the local authority. Over the last 25 years, Stockport has had the same Director of Public Health, Dr Stephen Watkins, appointed in 1990. Stockport has resisted demands to merge and reorganise on the basis that there are huge benefits to be gained from remaining coterminous. The sharing of boundaries has facilitated collaborative working and allowed health to remain at the centre of local authority proceedings (Jones and Pickstone 2008: 114). Notably the consistency in strategy enabled by organisational continuity has produced measurable benefits for the local population as Stockport’s health has improved from ‘being slightly worse’ than the national average to ‘slightly better’ than the national average over the period (Local Authority Vital Statistics 1974–2006).

## Learning from This Past

What does this history say about today’s plans for devolution? There has only been space to provide glimpses into the rich and complex history of health organisation in GM but it is clear that the challenges of delivering healthcare across multiple geographies and communities with persistent health inequalities have remained constant since the industrialisation of Manchester and its surrounding areas in the nineteenth century. The story of the rise and fall of mental health services in Salford during the 1950s and 1960s speaks to the dynamic nature of relations between health organisations, local and national politics and individual agency. At a time when NHS structures did not work in favour of linking hospital and community services, the combined efforts of Burn, Susser, Freeman and other staff supported by the Council and enabled by funding achieved change that improved the continuity in care and community support services for patients. Yet the service rapidly disintegrated because of a combination of multiple factors including change in local politics, the national reorganisation of social services and the loss of key personnel. Success in North Manchester during the 1980s was achieved despite economic pressures and strong national directives on rationalising services. The significant gains can be seen as the consequence of the particular ambitions and abilities of Moore, Leeson and other members of the team to focus on local needs and work collaboratively towards meeting these. The stability in local structures and continuity in personnel were undoubtedly instrumental. Developing new services around chronic diseases such as diabetes, sickle-cell anaemia and thalassaemia in Central Manchester during the 1980s and 1990s succeeded because of a combination of social, political and medical factors. Tomlinson was willing and capable of engaging with the Conservative mind set of management and efficiency savings and committed to developing local diabetes services to meet new national standards of care. His appointment of Pooley and his willingness to take on board her focus on patient needs shaped the service which was responsive to the particular needs of local ethnic communities. The creation of the Sickle-Cell and Thalassaemia Centre is a story of close attention to local need and collaboration with local communities built on long-standing efforts to improve local social cohesion. The chequered history of citywide working shows that there has been no lack of willingness to build effective networks for delivering health improvements across GM. Yet it is equally evident that throughout the period the short-term cycles of political change which have brought constant reorganisation and shifts in policy have been to the detriment of local ambitions. Improvements have been easier to achieve in places like Stockport and Salford where co-terminosity has been a benefit.

Map these issues on to the proposals for ‘Devo Manc’ and what can we say? Building a shared agenda across health, social care, voluntary and community organisations will be critical and will depend on local champions who have the ability to create a shared vision and work collaboratively. Creating deep understandings of the diverse needs and priorities of local health populations and harmonising these at the different levels of service delivery will be key. The most positive take-home message is that history shows that improvements are possible and although they can be enabled by economics and structures, they are not dependent on them. The caveat is that successful configurations are sensitive to destabilisation, most frequently from national policy and political shifts. ‘Devo Manc’ offers an opportunity to build on the achievements in GM’s health history but it will need stability if it is to succeed.
